# Effect of pH, Ionic Strength and Agitation Rate on Dissolution Behaviour of 3D-Printed Tablets, Tablets Prepared from Ground Hot-Melt Extruded Filaments and Physical Mixtures

**DOI:** 10.3390/biomedicines11020375

**Published:** 2023-01-27

**Authors:** Nour Nashed, Stephanie Chan, Matthew Lam, Taravat Ghafourian, Ali Nokhodchi

**Affiliations:** 1 Pharmaceutics Research Laboratory, Arundel Building, School of Life Sciences, University of Sussex, Brighton BN1 9QJ, UK; 2 Department of Chemical and Pharmaceutical Sciences, School of Human Sciences, London Metropolitan University, 166-220 Holloway Road, London N7 8DB, UK; 3 Department of Pharmaceutical Sciences, College of Pharmacy, Nova Southeastern University, 3200 South University Drive, Fort Lauderdale, FL 33328, USA; 4 Lupin Inhalation Research Center, 4006 NW 124th Ave., Coral Springs, FL 33065, USA

**Keywords:** 3D printing, hot-melt extrusion, physical mixture, biodissolution, ionic strength, agitation rate

## Abstract

With the current focus on 3D-printing technologies, it is essential to understand the processes involved in such printing methods and approaches to minimize the variability in dissolution behaviour to achieve better quality control outcomes. For this purpose, two formulations of theophylline tablets were prepared using hydroxypropyl cellulose (HPC) and ethyl cellulose (EC). Among the two types of tablets, three different methods (physical mixture (PM), hot-melt extrusion (HME) and 3D-printing fused deposition modelling (FDM)) were applied and their dissolution behaviours were studied under various conditions using a biodissolution tester. This was carried out at pH values of 1.2, 2.2, 5.8, 6.8, 7.2 and 7.5, mimicking the medium in the gastrointestinal tract. Dissolution tests under two dipping rates (10 dpm and 20 dpm) and two ionic strengths (0.2 M and 0.4 M) were conducted to mimic fed and fasting conditions. The dissolution efficiency (DE%), release rate, similarity factor (*f*_2_) and difference factor (*f*_1_) were calculated. When comparing the DE%, the formulation containing EC showed less sensitivity to changes in the dipping rate and ionic strength compared to the HPC formulation. As for the manufacturing method, 3D-printing FDM could improve the robustness of the dissolution behaviour of both formulations to dipping rate changes. However, for ionic strength changes, the effect of the manufacturing method was dependent on the formulation composition. For example, the 3D-printed tablets of the HPC formulation were more sensitive to changes in ionic strength compared to the EC-containing formulation. The release mechanism also changed after the thermal process, where *n* values in the Korsmeyer–Peppas model were much higher in the printing and HME methods compared to the PM. Based on the formulation composition, the 3D-printing method could be a good candidate method for tablets with a robust dissolution behaviour in the GI tract. Compared to HPC polymers, using hydrophobic EC polymers in printable formulations can result in a more robust dissolution behaviour in fed and fasting states.

## 1. Introduction

The dissolution behaviour of tablets in the gastrointestinal (GI) tract is a critical step in their absorption and biological response. Many factors can influence the dissolution of tablets, including those related to GI tract conditions and those controlled by drug properties, tablet formulation and manufacture characteristics. For example, the wide range of pH values in the GI tract (between 1.2 and 7.5) can affect drug solubility and polymer behaviour, hence effectively influencing drug release [1]. In addition, based on the health condition of the GI tract and the food intake, hydrodynamic forces and ionic strength may change, which can affect drug release [1,2]. Furthermore, an increase in the motility of the stomach and intestines can promote the disintegration of tablets and boost the drug release. The increased secretion of ions when consuming food can cause salting in/out of polymers and change the drug release behaviour of tablets [3,4]. Such GI factors can be simulated in vitro by adjusting the agitation speeds and dissolution media components during dissolution testing, in order to achieve a better prediction of the tablet dissolution behaviour in vivo. 

The dissolution test is an in vitro method used as a quality control tool which, based on the applied conditions, could be sensitive to and detect changes in the formulation composition and critical product attributes [5,6]. It is also needed for bioequivalence studies and in vitro–in vivo correlation (IVIVC) studies when developing new formulations. An effective dissolution test should closely simulate the drug release in the GI tract. Therefore, for more predictive in vitro results, it is preferable to use a series of conditions that mimic the environment of the GI tract in various physiological states [7,8]. A comparison of the release profiles obtained under various dissolution conditions can be used as a robustness indicator to show if the tablet will behave similarly under various physiological conditions. In the case of highly soluble drugs such as BCS I and III, simplified dissolution conditions could be used; however, a more sophisticated test condition is needed for poorly water-soluble drugs [7]. 

Although it has been suggested that a biodissolution apparatus (USP 3) is preferable for studying the dissolution behaviour for extended-release oral formulations under fed and fasted conditions by manipulating the pH and ionic strength of the media and changing the agitation rate [9], it is still not as widely used as a paddle or basket dissolution apparatus in dissolution studies. The hydrodynamic stress in USP 3 is superior to that in USP 1 and 2, as the 5 dips per minute (dpm) speed in USP 3 matches a 50 rpm paddle speed in USP 2 and a 100 rpm basket speed in USP 1 [10]. In IVIVC studies, USP 3 facilitates the application of in vivo-like conditions, including a variety of physicochemical and hydrodynamic conditions such as pH gradients, ionic strengths and agitation speeds, giving more comprehensive and predictive in vitro results [11,12]. Many methodologies have been proposed for USP 3 with regards to studying dissolution profiles in in vivo-like conditions [13,14,15].

The manufacturing method is one of the critical product attributes that can affect a tablet’s characteristics, including its dissolution performance, hence the drug bioavailability [5]. For example, 3D printing using the FDM method involves the melting/softening of materials at high temperatures and extrusion, the products of which are hardened on cooling. This is very different from various conventional compaction tabletting methods. With the current increasing interest in 3D-printing technology for tablet manufacture, it is crucial to investigate the mechanisms involved in the process that impact the dissolution profile of such tablets. Dissolution profile changes after 3D printing may be associated with changes in the porosity, density and hardness, depending on the formulation and printing conditions [16,17]. Thus, a discriminatory dissolution testing method is needed to provide a better understanding of how the printing method can change the release behaviour of a given formulation, which will consequently influence the bioavailability. This work investigates how the printing method can change the release profile under harsh dissolution conditions such as high agitation rates and high ionic strengths. In the authors’ previous work [16], dissolution studies were carried out in USP 2, while in this paper, more harsh dissolution conditions were applied using a USP 3 apparatus to predict the in vivo behaviour of printed tablets. To the best of the authors’ knowledge, this is the first time a USP 3 apparatus has been utilized for a comparison study of the dissolution profile of printed tablets with tablets prepared by conventional manufacturing methods, either from physical mixtures of the formulation powders (PM) or from filaments obtained through the hot-melt extrusion of the same formulation (HME). For all these manufacturing methods, two formulations were prepared using HPC or EC as the polymer and theophylline as the active pharmaceutical ingredient. The tablets were manufactured by three different methods: PM, HME and 3D printing via FDM. The data from the drug dissolution tests of the mentioned production methods were compared amongst each other to evaluate the impact of variable dissolution conditions on the drug release behaviour of the tablets. The test was carried out in the biological pH range from 1.2 to 7.5, with different agitation speeds (dipping rate; 10 and 20 dpm) and ionic strengths (0.2 M and 0.4 M) to simulate fed and fasted states in the GI tract.

## 2. Materials and Methods

### 2.1. Materials

Theophylline anhydrous with a purity of >99% was purchased from Fisher Scientific (Loughborough, UK). Two grades of hydroxypropyl cellulose (HPC), i.e., klucelTM EF and klucelTM JF, were provided by Ashland Inc. (Rotterdam, Netherlands). Ethyl cellulose (EC, Ethocel 10 FP) was obtained from Colorcon Ltd. (Dartford, UK). Dibutyl sebacate (DBS) was supplied by Sigma-Aldrich (St. Louis, MO, USA). Dissolution buffer solutions were prepared using the following materials: hydrochloric acid (HCl), potassium chloride (KCl), sodium hydroxide (NaOH) and potassium monobasic phosphate (KH2PO4), which were supplied by Fisher Scientific (Loughborough, UK), and deionised water. All materials were used as received. 

### 2.2. Tablet Formulations

Two formulations were prepared (Table 1) and were selected for biodissolution studies. The theophylline percentage was fixed at 30% in both formulations, and the remainder was polymers (e.g., EC and HPC), as shown in Table 1. Two viscosity grades of HPC were used, EF (low viscosity grade) and JF (high viscosity grade). The selection of the HPC grade was based on the printability of filaments. EC was plasticised with 5% DBS, which was left overnight for better sorption of the plasticizer into the polymer matrix. HPC, EC and theophylline were selected because they are thermally stable materials under the used conditions [16]. The formulations were converted to tablets by three different manufacturing methods, which are described in Section 2.3 below.

### 2.3. Preparation Methods of Tablets

The two formulations were prepared via three different methods: compression of a physical mixture of powders (PM), compression of hot-melt extruded powders (HME), and 3D printing of the powders (FDM). The average weight of the tablets prepared by any of the three methods was 333.33 mg, where theophylline accounted for 100 mg. The tablets were white in colour, round and cylindrical. They had the same surface area-to-volume ratio (SA/V) of 0.8 mm^−1^ to minimize its effect on the dissolution behaviour. 

#### 2.3.1. Preparing Physical Mixture (PM) Tablets 

Based on the composition mentioned in Table 1, the powders were thoroughly mixed using a mortar and pestle (for approximately 5 min). The physical mixture powder blend was compressed into a tablet (333.33 mg) with a manual tableting press (Model MTCM-ɪ, Globe Pharma, USA) equipped with 10 mm-diameter concave punches. The employed pressure was 150 bar and the dwelling time was adjusted to 10 s. The manufactured tablets were stored in enclosed vials in a cabinet at room temperature (22 ± 2 °C) and tested for dissolution within a week. 

#### 2.3.2. Preparing HME Tablets 

The powders were mixed manually and fed to a 10 mm twin-screw extruder L/D 20 (assembled by Point 1 Control Systems Ltd., Stoke-on-Trent, UK) at a screw speed of 50 rpm. The temperature of the extruder was set to over the Tg of the used polymers (around 120–130 °C) so that they were softened and mixed properly with the theophylline to obtain smooth and cylindrical filaments. The temperature of the feeding zone was 110 °C and that of the other sections (including the die) was 150 °C. The filaments were collected using a winder manually and were ground after being cooled down using a ball mill (PM 100, Retsch GmbH Germany) at 400 rpm for 4 min. The resulting powder was finally compressed into tablets using a manual tableting press, as with the PM tablets (see Section 2.4 for details) at a pressure of 150 bar and 10 s of dwelling time. The tablets produced were stored in enclosed vials in a cabinet at room temperature and tested for dissolution within a week.

#### 2.3.3. Preparing 3D-Printed Tablets 

Filaments produced by HME (see Section 2.3.2 for details) were used as feedstock for 3D printing, with a diameter between 1.6 and 1.8 mm to fit the printing nozzle (1.75 mm). The tablets were printed with a height of 5.45 mm and a diameter of 8.8 mm. These specific dimensions of the 3D-printed tablets matched the weight (333.33 mg) as well as the SA/V of the tablets prepared by HME and PM, which increased the accuracy when comparing their drug-release profiles. The printing settings were as follows: a layer height of 0.2 mm, an infill density of 100%, a number of shells of 2, a printing speed of 90 mm/s, a bed temperature of 50 °C and a nozzle temperature of 220 °C. The produced tablets were collected and stored in enclosed vials in a cabinet at room temperature and tested for dissolution within a week.

### 2.4. Biodissolution Studies

An automated USP 3, Bio-Dis (Varian, US), was used to carry out the dissolution tests. The dissolution test was performed in a varied range of pH values, covering the pH values in the GI tract, using buffered solutions, as shown in Table 2. The dissolution program was employed as reported in other studies [18,19]. In brief, tablets were kept for 60 min at a pH of 1.2, followed by 60 min at a pH of 2.2, then 10 min at a pH of 5.8, 120 min at a pH of 6.8, 30 min at a pH of 7.2 and 30 min at a pH of 7.5. The total duration was 310 min. The absorbance of the released theophylline was measured at a wavelength of 271 nm using a UV/Visible spectrophotometer (Varian, Cary 50). Two dissolution variables were tested, the dipping rate and the ionic strength. The dissolution data were analysed to explore which manufactured method could produce tablets that were robust enough in terms of drug release when subjected to fasted and fed states in the GI tract. For this purpose, the dissolution efficiency (DE%), difference factor (*f*_1_) and similarity factor (*f*_2_) were calculated for a quantitative comparison. The DE% is the area under a dissolution curve between defined time points.

$$f_{1}=\frac{\sum_{j=1}^{n}\left|R_{j}-T_{j}\right|}{\sum_{j=1}^{n}R_{j}}\times 100$$

$$f_{2}=50\times \log\left\{{\left[1+\left(1∕n\right)\overset{n}{\underset{j=1}{\sum }}{\left|R_{j}-T_{j}\right|}^{2}\right]}^{-0.5}\times 100\right\}$$

In the above equations, *R_j_* and *T_j_* are the mean percentages of the drug dissolved at each time point for the reference and test products, respectively. *n* denotes the number of samples taken during the dissolution run. When *f*_2_ was above 50% and *f*_1_ was less than 15%, there was no difference between the two dissolution profiles compared. 

#### 2.4.1. Dipping Rate

The dissolution test was run according to the proposed program at a low speed (10 dpm). The tablets were also tested for dissolution at a higher dipping rate of 20 dpm. The buffer solutions with pH values ranging between 1.2 and 7.5 were used as the dissolution medium for both of the mentioned dipping rates. During the study on the dipping rate, the ionic strength was fixed to the characteristic ionic strength values for each pH value as per Table 2 (0 M added of KCl). Changes in the dipping rate mimicked the changes in GI motility under various physiological states and during fed and fasted states. 

#### 2.4.2. Ionic Strength

To the various buffers listed in Table 2, KCl was added in two concentrations of 0.2 M and 0.4 M to study how ionic strength changes can affect drug release. This simulated the ionic strength changes as the result of food presence in the GI tract [3,14,18,19]. Note that each buffer in Table 2 has its own pH-specific ionic strength, but the focus here will be on the effect of the further added ionic strength (by adding KCl) to each buffer solution. The dissolution results in buffers without any additional ionic strength were considered as the reference to compare with the results after adding 0.2 M and 0.4 M ionic strength. During the study of ionic strength, the dipping rate was fixed at 10 dpm. The ionic strength was calculated using the equation below:

$$I=\frac{1}{2}\;\overset{j}{\underset{i}{\sum }}C_{i}Z_{i}^{2}$$

In the above equation, *I* is the ionic strength, *j* is the number of species of ions in the solution, *C_i_* is the molar concentration of ion *i* to *j* and *Z_i_* is the charge number of ion *i*.

### 2.5. Release Kinetics

The release kinetics for all the tablets prepared by the three methods were analysed using DDSolver software (an add-in program in Microsoft Excel) [20]. Data were fitted to the Korsmeyer–Peppas model, while special attention was paid to the *n* values of this equation in order to explore any change in the release mechanisms when the tablets were subjected to different dipping rates and ionic strengths. Below is the Korsmeyer–Peppas model, where Mt/M∞ is the fraction of the drug released at time t, K is the drug release rate constant and *n* is the release exponent:

Mt/M∞ = Kt*^n^*

The values of the diffusional exponent, *n*, differ based on the geometry of the tablet. For 60% of the release profile of a cylindrical-shaped tablet, the drug release is governed by diffusion (Fickian model) when *n* approaches 0.45. However, when *n* approaches 0.85, swelling (polymer chain relaxation) is the main release mechanism, where the drug release follows zero-order kinetics (non-Fickian model, case II). For 0.45 < *n* < 0.85, the drug release is governed by both diffusion and swelling (anomalous transport). Values of the diffusional exponent, *n*, exceeding 0.85 mean that the drug release is controlled by polymer relaxation (non-Fickian model, super case II) [21].

## 3. Results and Discussion

### 3.1. Effects of Dipping Rate and Formulation Composition 

**Figure 1:**
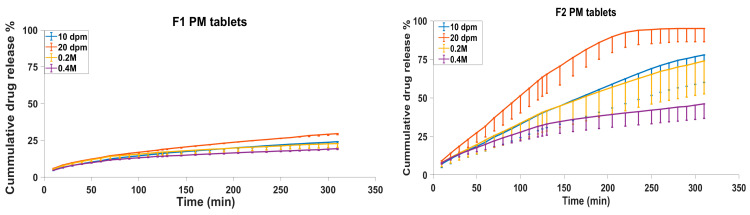

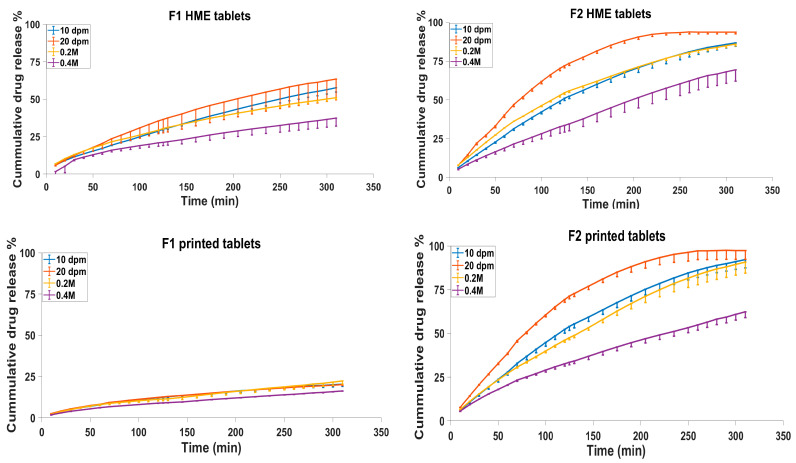
Dissolution profiles of F1 and F2 tablets prepared by the three manufacturing methods (PM, HME and 3D-printed) measured under dipping rates of 10 dpm and 20 dpm, and added ionic strengths of 0.2 M and 0.4 M. Error bars indicate SD values. For the clarity of figures, error bars were drawn from just one side.

Dissolution tests were carried out in three replicates for all PM, HME and 3D-printed tablets of both formulations under different dipping rates (10 dpm and 20 dpm) and added ionic strengths (0.2 M and 0.4 M). The dissolution profiles are depicted in Figure 1. The dissolution results at 10 dpm in different pH media were considered as the reference, which was used to investigate the effects of a higher agitation rate (20 dpm) and added ionic strength (0.2 and 0.4 M) on the dissolution rate of the tablets manufactured via three different methods. The DE% values were used for the overall comparison of the dissolution behaviour, and the release rate calculated for each pH value was used for an in-depth understanding of the release behaviour and also to determine how the formulation composition and manufacturing methods affected the dissolution behaviour of tablets. The release rate is usually calculated in two different ways, either from the release% vs. time plot or from the release% vs. square root of the time plot [6,22]. As the release% versus time showed higher R^2^ values compared to when the release% was plotted versus the square root of time, the former was used to calculate the release rate at each pH value.

Figure 1 shows that tablets prepared by PM and HME had higher SD values compared to the printed tablets, implying that the printing method could improve the reproducibility of the dissolution behaviour, as shown by the reduced variations from batch to batch. The reason behind this is not clear, but the increased hardness of the printed tablets compared to the HME and PM tablets, as proven in our previous work, could have had an impact [16].

Looking at the dissolution graphs in Figure 1, it is clear that the F1 formulation had a much slower release pattern compared to F2, regardless of the manufacturing method and dissolution conditions. Under the 10 dpm dissolution testing condition, the DE% values of F1 were 16.57%, 33.76% and 13.12% for the PM, HME and printed tablets, respectively, while F2 showed a faster release with DE% values of 45.32%, 53.91% and 57.12% for the PM, HME and printed tablets, respectively (Table 3). A similar pattern of a faster F2 compared to F1 release was seen under a 20 dpm dipping rate and the 0.2 M and 0.4 M added ionic strengths. This shows that the presence of the insoluble EC polymer in F1 made the tablet more hydrophobic than the F2 tablets, which may have been responsible for the slow drug release. 

From Figure 1, it can be deduced that thermal treatment plays a major role in the release of theophylline. This is obvious, since the dissolution profiles of both the heat-treated F2 tablets, i.e., the HME tablets made of extruded filaments and the 3D-printed tablets, showed faster dissolution rates than the PM tablets under various dissolution conditions. For example, the DE% of the HME and printed tablets of F2 at 10 dpm were 53.91 and 57.12%, while this was lower for the PM tablets at 45.32%. This was also observed in our previous work where distilled water was used as the dissolution medium [16]. The effect of heat on promoting the dissolution rate can be attributed to the fast cooling of the melted/softened material, leading to an increased free volume and better water penetration to amorphous polymers, as described in other studies [16,23,24]. Another possible explanation is that, during heat treatment, the theophylline particles highly disperse in the molten polymer matrix, and because theophylline is a water-soluble drug, this can improve water penetration to the polymer matrix and facilitate the drug release [3]. A similar pattern was seen for the F2 tablets in other dissolution conditions, i.e., the dipping rate of 20 dpm, and ionic strengths of 0.2 M and 0.4 M. With the F1 formulation, a similar effect of heat treatment was observed for the HME tablets, but the 3D-printed F1 tablets were anomalous with an unusually low DE%. 

Table 3 shows that the dissolution efficiency values (DE%) of the F1 formulation were increased when the agitation rate was increased from 10 dpm to 20 dpm for the PM and HME tablets, but this was not the case for the 3D-printed tablets. For example, the DE% increased from 16.57% to 19.82% in the case of the physical mixtures, whereas this value remained the same in the case of the 3D-printed tablets (around 13% at both agitation rates). This indicates that the 3D-printed F1 tablets were more robust against the agitation rate; hence, they were expected to produce a more consistent release profile in vivo under various GI motility rates. On the other hand, the 3D-printed F2 formulation showed some variability, where an increase in the agitation rate increased its DE% value to some extent. Despite this, the change in the dissolution efficiency was much smaller for the 3D-printed tablets compared with the HME and PM tablets, with percentage changes of 31, 22 and 19% for the PM, HME and 3D-printed tablets, respectively, when the agitation rate increased from 10 to 20 dpm. This once again shows that the 3D-printed tablets showed more resistance against the agitation rate compared to the tablets made from PM or HME.

Generally speaking, an increase in the DE% at a high agitation rate could be due to the depletion of the surface gel layer (diffusion layer) due to the agitation. The thickness of the surface diffusion layer is inversely correlated with the dissolution rate and its depletion is expected to increase the drug diffusion based on the Noyes–Whitney equation [1]. The depletion of the gel layer can also restrict the effect of gelling on controlling the drug release, making the drug release at 20 dpm faster than at a low agitation speed (10 dpm).

Table 3 also gives the similarity factors calculated for the same tablet tested at two different agitation rates. The similarity values show a significant difference in the dissolution profile of all F2 tablets compared to the insignificant change for all F1 tablets, indicating a higher sensitivity of the F2 formulations to the agitation rate compared to the F1 formulation. For example, the DE% of PM tablets with the F2 formulation increased from 45.32% at 10 dpm to 65.35% at 20 dpm, showing a significant difference in the dissolution profiles with a low similarity factor of *f*_2_ = 34.27%. On the other hand, the F1 formulation showed a much lower increase in DE%, from 16.57 to 19.82%, with a high similarity of dissolution profiles at various agitation rates with *f*_2_ = 72.27%. The high sensitivity of the F2 formulation could be due to the polymeric matrix containing only the hydrophilic polymer HPC, which is probably more susceptible to gel layer depletion [6] than the polymer matrix in F1, which has 35% of the non-swellable polymer, EC, in its composition. 

Overall, the 3D-printed F1 tablets have the highest *f*_2_ values when the release profiles are compared between the 10 and 20 dpm agitation rates (Table 3). This means that the 3D-printing method along with the use of EC improved the formulation robustness to agitation changes. This could be attributed to both the hydrophobic composition (EC) and the hard and solid structure of the printed tablets, making them more resistant to hydrodynamic mechanical stress. 

**Figure 2:**
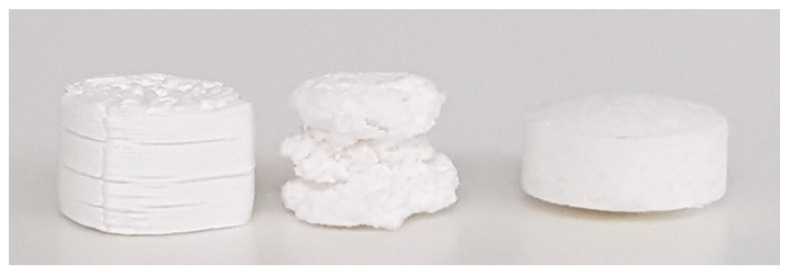
F1 tablets after dissolution test. From left to right: printed, HME and PM tablets. The three types of tablets had the same cylindrical geometry with the same SA/V (0.8 mm^−1^) before dissolution. However, the residual after dissolution (shown in this figure) showed that PM and printed tablets preserved their integrity while HME tablets showed erosion, which could be responsible for the increased release % of HME tablets compared to PM and printed ones, as shown in Figure 1.

The unexpectedly low DE% of the 3D-printed F1 tablets, which was also seen under different dipping rates (Table 3) and ionic strengths (Table 4), may be justified by the lack of disintegration of the 3D-printed tablets (Figure 2). Both the PM and printed tablets showed a higher integrity during dissolution compared to HME tablets, with slower disintegration. As proven in our previous work [16], the disintegration of HME tablets can be attributed to the high water uptake of this F1 formulation. Although the printed tablets were prepared from hot-melt extruded filaments, they showed no disintegration during the dissolution period, which also correlates with the high hardness of these tablets (>550 N), as reported in our previous work [16].

It is also notable that in the case of the F2 formulation under high agitation (20 dpm), the difference between the DE% values (Table 3) was not significant, with the DE% values varying between 65 and 69%. 

**Figure 3:**
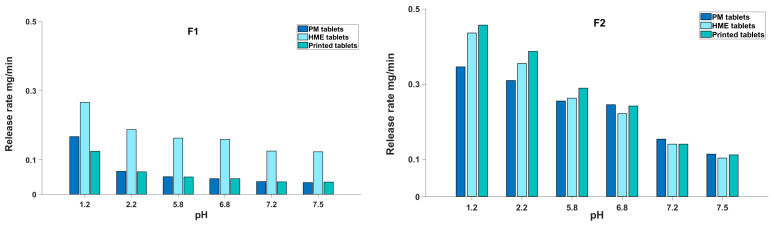
Bar charts comparing release rate at each pH medium during dissolution test at 10 dpm for PM, HME and printed tablets in both formulations.

Figure 3 shows the release rate of the various tablets at 10 dpm. The release rate was calculated using a zero-kinetics model that showed a good fit for the parts of the release profiles in each pH medium (R^2^ values > 0.99). The F1 tablets contained 35% of the hydrophobic polymer, EC, while F2 contained just HPC (Table 1). This difference in the polymer composition led to considerable differences in the release rates, as discussed earlier. Figure 3 shows that for F1, there was a burst release in the first hour of the test at a pH of 1.2, followed by more steadiness in the release rate for the rest of the dissolution run at increasing pH levels. For example, the HME tablets showed drug release rates of 0.27, 0.19, 0.16, 0.16, 0.13 and 0.12 mg/min at pH values of 1.2, 2.2, 5.8, 6.8, 7.2 and 7.5, respectively. However, F2 in general showed a burst release with a sounder gradual decrease in the release rate over time (Figure 3). For instance, the HME tablets started with a release rate of 0.44 mg/min as a burst release at a pH of 1.2, which then dropped to 0.36, 0.26, 0.22, 0.14 and 0.10 mg/mL as the pH increased to 2.2, 5.8, 6.8, 7.2 and 7.5, respectively. It must be noted that the solubility of theophylline, a very weak acid of pKa 8.81, was not expected to change considerably at pH variations [25]. 

The burst release, in the beginning, is likely to be driven by the release of the drug on the surface of the tablets. This is followed by a decrease in the release rate over time, which can be explained by two mechanisms as follows. First, in the case of water-soluble drugs such as theophylline, the release rate is related to the amount of drug available for dissolution, which decreases over time, causing the release rate to decrease [26]. The second reason is the decrease in the tablet’s surface area as a result of dissolution during the dissolution testing, which, according to the Noyes–Witney equation, can decrease the release rate by the end of the test [1].

### 3.2. Effect of Ionic Strength

The initial ionic strength of the buffer solutions was used as the reference for data comparison. A dipping rate of 10 dpm was used for the ionic strength study. Parameters extracted from the dissolution profiles recorded with modified ionic strengths are listed in Table 4 and Table 5. Data from Table 4 shows that an increased ionic strength due to the addition of 0.2 M KCl had no significant effect on the dissolution profiles of any of the tablets. However, according to Table 5, when the ionic strength was further increased by adding 0.4 M KCl, different dissolution profiles were observed for several types of tablets. 

Table 5 shows that, in general, an increase in the added ionic strength from 0 to 0.4 M caused a reduction in the release rate, resulting in a low similarity between the release profiles, as seen from the low *f*_2_ values for several tablet types. Furthermore, the DE% of formulation F2 was more sensitive to ionic strength changes compared to the F1 formulation. When the percentage change in the DE% as a result of the 0.4 M KCl addition was calculated for both the F1 and F2 formulations, the results showed that the changes in the DE% for F1 were 15, 25 and 27%, whereas these values for the F2 formulation were 32, 30 and 35% for the PM, HME and 3D-printed tablets, respectively. This indicates that the F2 tablets were not consistent when the composition and ionic strength of the media was modified. However, for F1, the high *f*_2_ values for the PM and 3D-printed tablets show the consistency of their release profiles when varying the dissolution media and a resistance to the increased ionic strength.

**Figure 4:**
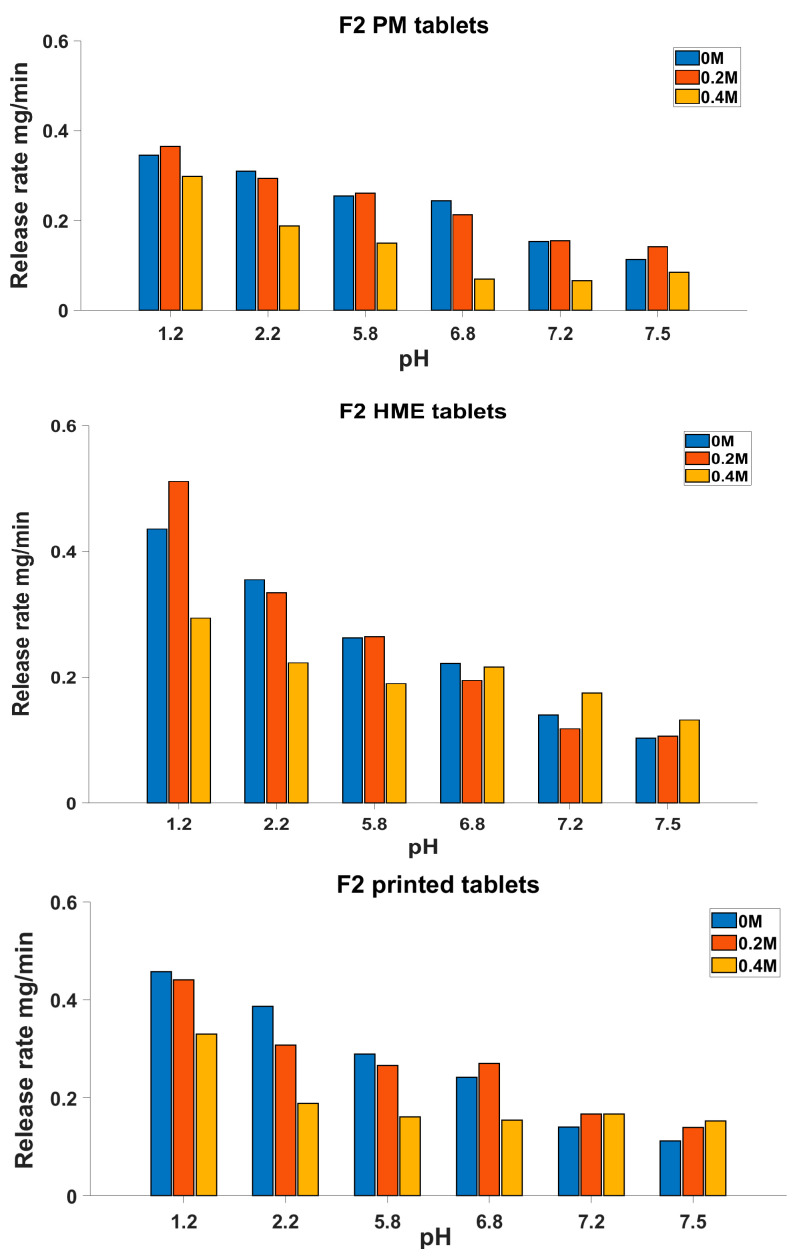
Release rate in each pH medium during dissolution test at different added ionic strengths for F2.

**Figure 5:**
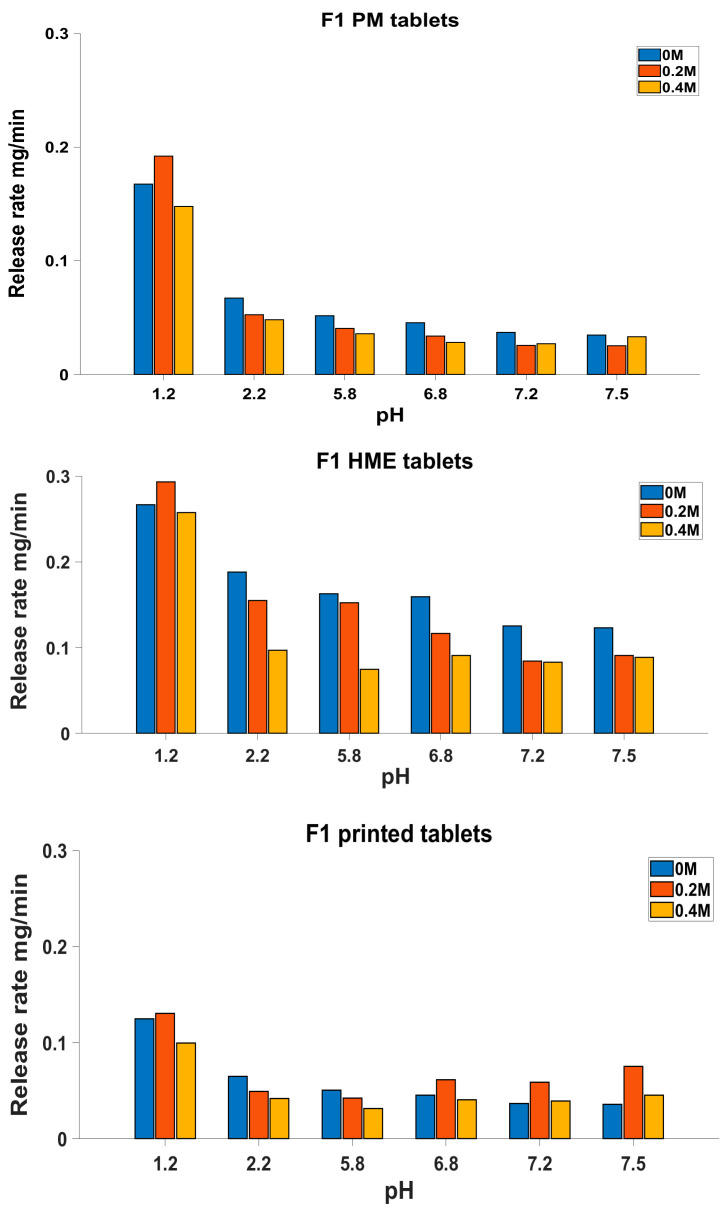
Release rate in each pH medium during dissolution test at different ionic strength additions for F1.

A more detailed comparison of the dissolution profiles can be seen in Figure 4 and Figure 5, where the dissolution rates at various pH values of the dissolution medium are illustrated. The release rates of the F2 tablets (Figure 4) showed an interesting pH-dependant pattern, where adding 0.2 M ionic strength led to an increase in the release rate at a pH of 1.2 and also at some higher pH values. This increase in the release rate could be justified by the effect of alkaline ions such as potassium on improving the solubility of theophylline [27]. 

Figure 4 shows that the release rate at a pH of 1.2 decreased when 0.4 M KCl was added. As a result of the 0.4 M KCL addition, in the first hour of dissolution at a pH of 1.2, the theophylline release rate of the F2 formulation was decreased from 0.35, 0.44 and 0.46 mg/min for the PM, HME and 3D-printed tablets to 0.30, 0.29 and 0.33 mg/min, respectively, with PM showing the least reduction. This observation is opposite to what was expected from the chaotropic effect of K +; however, considering the complexity of the formulation and manufacturing process, it is obvious that there are several other processes that may be more dominant in controlling the drug release than the mere pure drug factors. For example, the effect of KCl ions on the gelling and salting out of HPC polymers and water penetration into various manufactured tablets may be affected by the excess salt, which will need to be further investigated. In addition, it has been suggested before that a high ionic strength can reduce the drug release from certain matrix formulations by reducing the water available for drug transportation [18,28]. 

Increasing the concentration of ions in the medium can reduce the amount of free water molecules available for the polymer hydration, since ions will compete with the polymer for binding with water molecules, leaving the polymer with less hydration/water penetration than what is required for the drug release [3,29]. Two types of water are found within the hydrated polymer, bound water and free water. The bound water is responsible for gelling behaviour while the free water is responsible for the transportation of solutes and thus, drug release and polymer dissolution [28]. When ions are added, free water can be reduced while maintaining gelling behaviour and tablet integrity, which would decrease the drug release [3,4]. 

It is worth mentioning that the 3D-printing method increased the sensitivity of the F2 formulation to the 0.4 M added KCl, with this tablet showing the least similarity of its release profile at *f*_2_ = 32.89% (Table 5). This might be due to the free volume changes under the heating/fast cooling process that may change the swelling behaviour of HPC and make it more susceptible to the ionic strength salting-out effect [30]. 

The release rates with 0.4 M KCl can provide an understanding for how the preparation method may change the drug release behaviours of the formulation. As for F2, in the PM tablets, the release rate with 0.4 M KCl decreased over time, with 0.30, 0.19, 0.15, 0.07, 0.07 and 0.08 mg/min at pH values of 1.2, 2.2, 5.8, 6.8, 7.2 and 7.5, respectively. This is a similar pattern to its release in a baseline medium with no KCl (Figure 4). However, after extruding and 3D printing, the release pattern of the F2 formulation changed in the dissolution medium containing 0.4 M KCl (Figure 4) and the reductions in the release rate over time became much smaller. In the HME tablets, the release rate was 0.29 mg/min at a pH of 1.2, and it decreased to 0.22, 0.19, 0.22, 0.17 and 0.13 mg/min at pH values of 2.2, 5.8, 6.8, 7.2 and 7.5, respectively. Similarly, in the printed tablets, the release rate was 0.33 mg/min at a pH of 1.2, which then decreased to 0.19, 0.16, 0.15, 0.17 and 0.15 mg/min at pH values of 2.2, 5.8, 6.8, 7.2 and 7.5, respectively. This indicates some major physical changes after the thermal process and fast cooling, which was also seen by an increase in the free volume of the polymer and a decrease in the density following these processes reported earlier [16]. Other studies have also shown that with increased free volume, the water uptake will be higher and this will affect mostly the free water available for transportation rather than the bound water; hence, the gel’s ability to retard the drug release will be reduced, and higher release rates will be observed [28].

Similarly, the preparation method clearly affected the release behaviour of the F1 formulation (Figure 5). Although all tablets showed a decrease in the release rate at the increased ionic strength (0.4 M KCl), this decrease was more pronounced in the HME tablets. At a pH of 6.8, the HME tablets had a release rate of 0.16 mg/min when no KCl was added; this dropped to 0.09 mg/min (43% reduction) with 0.4 M KCl. However, the PM and 3D-printed tablets showed a lower reduction of 37% and 8%, respectively, in the release rate at this pH as a result of the 0.4 M KCl addition. Similarly, in a pH of 2.2, the release rate dropped by 28%, 48% and 35% for the PM, HME and 3D-printed tablets, respectively. Contrary to the PM and 3D-printed F1 tablets, the HME tablets with the F1 formulation showed disintegration during dissolution in 0 M KCl compared to 0.4 M, where the tablet integrity was preserved during dissolution. This high sensitivity of the HME tablets to ionic strength may be due to a change in the hydrophilic/hydrophobic balance between EC and HPC as a result of the hot-melt extrusion, a behaviour that was also seen in our previous work [16].

At high pH values such as 7.2 and 7.5, the 3D-printed tablets had an increase in their release rate by 5% and 25% with 0.4 M of added ionic strength compared to 0 M, which was absent in the PM and HME tablets, which had lower release rates at a high ionic strength (Figure 5). The same pattern was seen for 0.2 M KCl, where the release rate from the 3D-printed tablets, unlike that of the PM and HME tablets, increased by 60% and 122% at pH values of 7.2 and 7.5 compared to the release rate at 0 M KCl. This increase was also seen in the F2 formulations, both for the 3D-printed and HME tablets, which could be attributed to the thermal effect leading to a decreased density and an increased specific volume after the heating/cooling process, and this could also be associated with expanded voids within the printed matrix compared to the HME and PM tablets, as discussed in the authors’ previous work [16]. This increase in specific volume, in turn, could increase the water penetration, improving the theophylline solubility and release, especially in the presence of basic ions at higher pH values. The dissolution rate-increasing effect of 0.4 KCl at high pH values, which is unique to tablets with a heat-treated manufacture, may indicate a substantial change in the microstructure of the formulation due to the melting and fast cooling processes.

### 3.3. Release Kinetics

The Korsmeyer–Peppas model was applied to the dissolution data and its coefficients, K and *n*, were calculated. The model fit to all the data was excellent, with R^2^ values over 0.99. The resulting *n* values for all formulations are listed in Table 6. The diffusional exponent (*n*) is an indication of the mechanism of drug release [31], and its values can indicate how different dipping rates and ionic strengths affected the release mechanisms. If *n* < 0.45 (for tablet shape), the release mechanism is through the diffusion of the drug out of the matrix according to the concentration gradient. This is the Fickian diffusion method, where solvent diffusion is less impactful than polymer chain mobility. If *n* approaches 0.85, it means that swelling/gelling behaviour governs the release of the drug and the main mechanism of drug release is erosion. In this case, polymer relaxation happens and the chain mobility is slower than water diffusion. Anomalous diffusion for 0.45 < *n* < 0.85 happens when the water mobility in the matrix allows diffusion based on the concentration while swelling is happening and controlling the release. Therefore, in anomalous mechanisms, both diffusion and swelling/dissolution control the drug release [32]. It follows that any change in swelling behaviour can change the *n* value. Since the tablets prepared in this study had similar geometries in terms of their surface area and shape, the changes in the *n* values would have been driven by changes in the formulation under extrusion and high temperatures or by dissolution conditions. 

Table 6 shows that increasing the dipping rate could increase the *n* values to some extent, especially for F2. This is probably because of the higher depletion of the surface gel layer, making erosion of the swelled layer more dominant for the drug release. On the other hand, the *n* values decreased with increasing ionic strength. The F1 HME tablets had an *n* value of 0.687 at 0 M KCl, which decreased to 0.587 and 0.553 with 0.2 M and 0.4 M KCl, respectively. Studies have shown that the more the swelling controls the release mechanism, the higher the *n* value is [32], which means that the swelling/erosion role in the drug release decreases under high ionic strengths. This claim agrees with the effect of the ionic strength on the swelling behaviour by reducing the free water available for drug transport within the gel/swollen layer [3,29].

In terms of the effect of the manufacturing method, changes in the *n* values showed that the thermal process of formulations can make the swelling/erosion influence sounder on the release mechanism. For example, the release mechanism of F2 moved from anomalous with *n* = 0.733 in the PM tablets to super case II after 3D printing with *n* = 0.873 (near zero-order release). This could have resulted from the increase in free volume. Water sorption can be faster and higher due to an increased free volume under the heating/cooling process, while 3D printing allows swelling to be the main release mechanism [16,32]. The same pattern was observed at 20 dpm (Table 6). Another example is F1 in a high-ionic-strength medium (0.4 M KCl). Without any thermal process, the PM tablets had Fickian diffusion (with *n* = 0.389), meaning the swelling effect was minimal, probably due to the effect of KCl ions on polymer dehydration. However, the drug release mechanism for the 3D-printed tablets became anomalous with *n* = 0.613, meaning the formulation underwent physical changes under extrusion and high temperatures that made the swelling/erosion mechanism more effective due to the thermal process. It can be concluded that the manufacturing method, and also the dissolution conditions, can alter the mechanism of drug release from diffusion to erosion or vice versa.

The 3D printing of tablets can introduce, as a manufacturing method, several physical changes that impact the release profile of the same formulation made into tablets by conventional tableting methods. Few comparison studies have been performed in this area, and changes in geometry, hardness, porosity and true density were not included in the dissolution data interpretation [17,32]. Thus, the authors have carried out more in-depth and integral comparison studies in their previous work, including all aforementioned factors [16]. The FDM printing method was compared to the HME and PM methods, and it was found that the printed tablets had the highest hardness and porosity, as discussed in this paper. In addition, the authors proved the decreased true density in printed tablets because of the fast cooling process, distinguishing their study from other pharmaceutical comparison studies. The current work gives a deeper understanding of how the FDM 3D-printing method impacts a formulation’s robustness to dissolution conditions, which is an extension of the authors’ previous work [16]. Overall, increasing the dipping rate can make the drug release faster, while increasing the ionic strength can achieve the opposite. Introducing a hydrophobic polymer, EC, seems to reduce the formulation’s sensitivity to dissolution variables. The influence of the decreased true density in printed tablets—associated with an increased free volume as discussed previously—on the release profile was obvious in alkaline buffers. As mentioned in other studies [28], the increase in free volume will allow more free water in the matrix, which can justify a higher release rate for hydrophilic formulations (containing HPC) in the printed tablets than in non-thermally processed tablets (PM ones). Changes in the release kinetics after printing can be also linked to a free volume increase (true density decrease) after thermal printing. When a water-swellable polymer is used, such as HPC in this study, water penetration can be higher due to an increased free volume induced by the heating/fast cooling process. This can cause the release mechanism to be controlled more by swelling/erosion, giving a high diffusional exponent (*n* value) in the Korsmeyer–Peppas model. This study shows how both the formulation composition and manufacturing method affect a tablet’s robustness to dissolution behaviour, which helps when developing robust tablets for in vivo dissolution conditions based on the used tableting method.

## 4. Conclusions

The effect of three tablet manufacturing methods, i.e., conventional tablet compression from a physical mixture, compression of hot-melt extruded (HME) filaments, and 3D FDM printing, on the release of theophylline measured under simulated fed and fasted conditions was studied using a biodissolution tester (USP 3 apparatus). The dissolution efficiency (DE%) increased by increasing the dipping rate, regardless of the formulation composition, while raising the ionic strength beyond a certain level decreased the DE%, indicating the effect of KCl ions on salting out the swellable polymers, and hence prolonging the drug release. Despite having similar drug-release patterns, the hydrophilic formulation with just the HPC polymer was highly sensitive to changes in the dipping rate and ionic strength compared to the formulation containing both HPC and the hydrophobic polymer, EC. The hydrophilic formulation also showed a high variability in the dissolution behaviour under different agitation rates and ionic strengths, indicating that under in vivo conditions, this formulation will probably be susceptible to changes under fed and fasted states in the GI tract, while the EC-containing formulation was more robust to variable dissolution conditions. The manufacturing method also impacted the formulation’s dissolution profile and its variability under different dissolution conditions. When EC was included in the formulation, the 3D-printing and conventional tableting methods produced more consistent dissolution profiles under variable dissolution conditions compared to the HME method. However, the 3D-printing method increased the variability of the dissolution profile as a result of the KCl addition when the hydrophilic formulation was used, indicating possible variable release behaviour in vivo under different fed and fasted conditions. This observation is mostly a formulation-related effect rather than a 3D-printing manufacturing effect, since the other two manufacturing methods also produced similarly high variability upon a 0.4 M KCl addition. Thus, the 3D-printing method seems to be preferable for hydrophobic formulations, and it produces a consistent release profile under different agitations and ionic strengths. The release mechanism was also dependent on the manufacturing method, where the thermal treatment in both the 3D-printing and HME methods could significantly increase the *n* values of the Korsmeyer–Peppas model. The results prove that the method of manufacturing is as critical as the composition in the design of robust formulations with consistent release profiles under various physiological conditions.

## Figures and Tables

**Table 1:** Composition of studied formulations.

Formulation **	Theophylline (% *w*/*w*)	HPC EF (% *w*/*w*)	HPC JF (% *w*/*w*)	EC (% *w*/*w*)
F1	30%	---	35%	35% *
F2	30%	70%	---	---

** Tablet weight was 333.33 mg, containing 100 mg of theophylline. * Plasticised with 5% *w*/*w* of DBS overnight.

**Table 2:** Composition, pH values and transit time of each buffer used in biodissolution test.

Mimicked GI Tract Segment	Buffer Composition *	Ionic Strength **	pH Value	Transit Time (min)
Stomach	0.05 M KCl and 0.073 M HCl in DW	0.12 M	1.2	60
Stomach	0.05 M KCl and 0.007 M HCl in DW	0.06 M	2.2	60
Duodenum	0.004 M NaOH and 0.05 M KH_2_PO_4_ in DW	0.28 M	5.8	10
Jejunum	0.022 M NaOH and 0.05 M KH_2_PO_4_ in DW	0.3 M	6.8	120
Proximal ileum	0.035 M NaOH and 0.05 M KH_2_PO_4_ in DW	0.3 M	7.5	30
Distal ileum	0.04 M NaOH and 0.05 M KH_2_PO_4_ in DW	0.32 M	7.8	30 min

* DW = distilled water. ** These ionic strengths are the initial ionic strength of the dissolution medium without adding more ionic strength to investigate the effect of ionic strength on dissolution behaviour.

**Table 3:** Parameters extracted from dissolution profiles, including DE% values and similarity (*f*_2_) and difference (*f*_1_) factors of the dissolution profiles of the various tablets dissolved at different dipping rates.

Formulation/Method	DE% *		*f*_2_	*f*_1_	Inference
	10 dpm	20 dpm			
F1 PM tablets	16.57	19.82	72.27	16.45	Similar
F1 HME tablets	33.76	39.14	62.10	13.67	Similar
F1 printed tablets	13.12	13.33	99.08	1.93	Similar
F2 PM tablets	45.32	65.35	34.27	29.96	Different
F2 HME tablets	53.91	68.90	40.36	21.52	Different
F2 printed tablets	57.12	69.41	44.71	17.53	Different

* No added KCl in dipping rate studies. Ionic strength was from the buffer composition (baseline ionic strength).

**Table 4:** DE% values obtained from dissolution testing performed with a dipping rate of 10 dpm at two different ionic strengths, the baseline ionic strength (0 M KCl added) and 0.2 M added KCl, along with similarity (*f*_2_) and difference (*f*_1_) factors for these two profiles.

Formulation/Method	DE%		Similarity Factor	Difference Factor	Inference
	0 M KCl	0.2 M KCl			
F1 PM tablets	16.57	17.05	85.19	6.91	Similar
F1 HME tablets	33.76	32.48	73.62	8.16	Similar
F1 printed tablets	13.12	13.03	94.55	4.98	Similar
F2 PM tablets	45.32	44.27	80.25	4.10	Similar
F2 HME tablets	53.91	55.76	76.46	4.20	Similar
F2 printed tablets	57.12	54.01	70.97	5.83	Similar

**Table 5:** Dissolution efficiency values (DE%) obtained from dissolution testing performed with a dipping rate of 10 dpm using buffers with two different ionic strengths, the baseline ionic strength (with 0 M KCl added) and with 0.4 M KCl added, along with the similarity (*f*_2_) and difference (*f*_1_) factors for these two dissolution profiles.

Formulation/Method	DE%		Similarity Factor	Difference Factor	Inference
	0 M KCL	0.4 M KCL			
F1 PM tablets	16.49	14.07	75.98	17.02	Similar
F1 HME tablets	33.66	23.03	45.63	32.11	Different
F1 printed tablets	13.08	9.85	72.77	24.52	Similar
F2 PM tablets	45.2	31.6	37.90	30.04	Different
F2 HME tablets	53.81	39.27	40.83	26.8	Different
F2 printed tablets	57.12	36.97	32.89	53.71	Different

**Table 6:** The Korsmeyer–Peppas *n* values for drug release from F1 and F2 tablets under different dissolution conditions of dipping rate (10 or 20 dpm) and added KCl concentrations.

Formulation/Method	10 dpm, 0 M KCl	20 dpm, 0 M KCl	10 dpm, 0.2 M KCl	10 dpm, 0.4 M KCl
F1 PM tablets	0.469	0.469	0.391	0.389
F1 HME tablets	0.687	0.716	0.587	0.553
F1 printed tablets	0.606	0.582	0.642	0.613
F2 PM tablets	0.733	0.765	0.687	0.527
F2 HME tablets	0.844	0.913	0.771	0.767
F2 printed tablets	0.873	0.907	0.791	0.692

## Data Availability

Not applicable.
